# Supplementary material for: Reprogramming of the transcriptome after heat stress mediates heat hormesis in Caenorhabditis elegans
Source: Nat Commun. 2023 Jul 13;14:4176. doi: 10.1038/s41467-023-39882-8 (PMC10345090; doi:10.1038/s41467-023-39882-8)
Supplement: Supplementary file 1 — Supplementary Information [file 41467_2023_39882_MOESM1_ESM.pdf]

Supplementary Information

Reprogramming of the transcriptome after heat stress mediates heat hormesis in Caenorhabditis elegans

Fan Xu[1, 2], Ruoyao Li[1], Erika D v. Gromoff[1], Friedel Drepper[3], Bettina Knapp[3], Bettina Warscheid[3, 4, 6], Ralf Baumeister[1, 2, 4, 5] and Wenjing Qi[1*]

1. Bioinformatics and Molecular Genetics (Faculty of Biology), Albert-Ludwigs-University Freiburg, Freiburg 79104, Germany
2. Spemann Graduate School of Biology and Medicine (SGBM), Albert-Ludwigs-University Freiburg, Freiburg 79104, Germany
3. Biochemistry-Functional Proteomics, Institute of Biology II, Faculty of Biology, Albert-Ludwigs-University Freiburg, Freiburg 79104, Germany
4. Signalling Research Centers BIOSS and CIBSS, Albert-Ludwigs-University Freiburg, Freiburg 79104, Germany
5. Center for Biochemistry and Molecular Cell Research (Faculty of Medicine), Albert-Ludwigs-University Freiburg, Freiburg 79104, Germany
6. Biochemistry II, Theodor Boveri-Institute, Biocenter, University of Würzburg, 97074 Würzburg, Germany

*Correspondence: wenjing.qi@biologie.uni-freiburg.de
Bio3/Bioinformatics and Molecular Genetics, University of Freiburg, Schänzlestrasse 1, D-79104 Freiburg, Germany

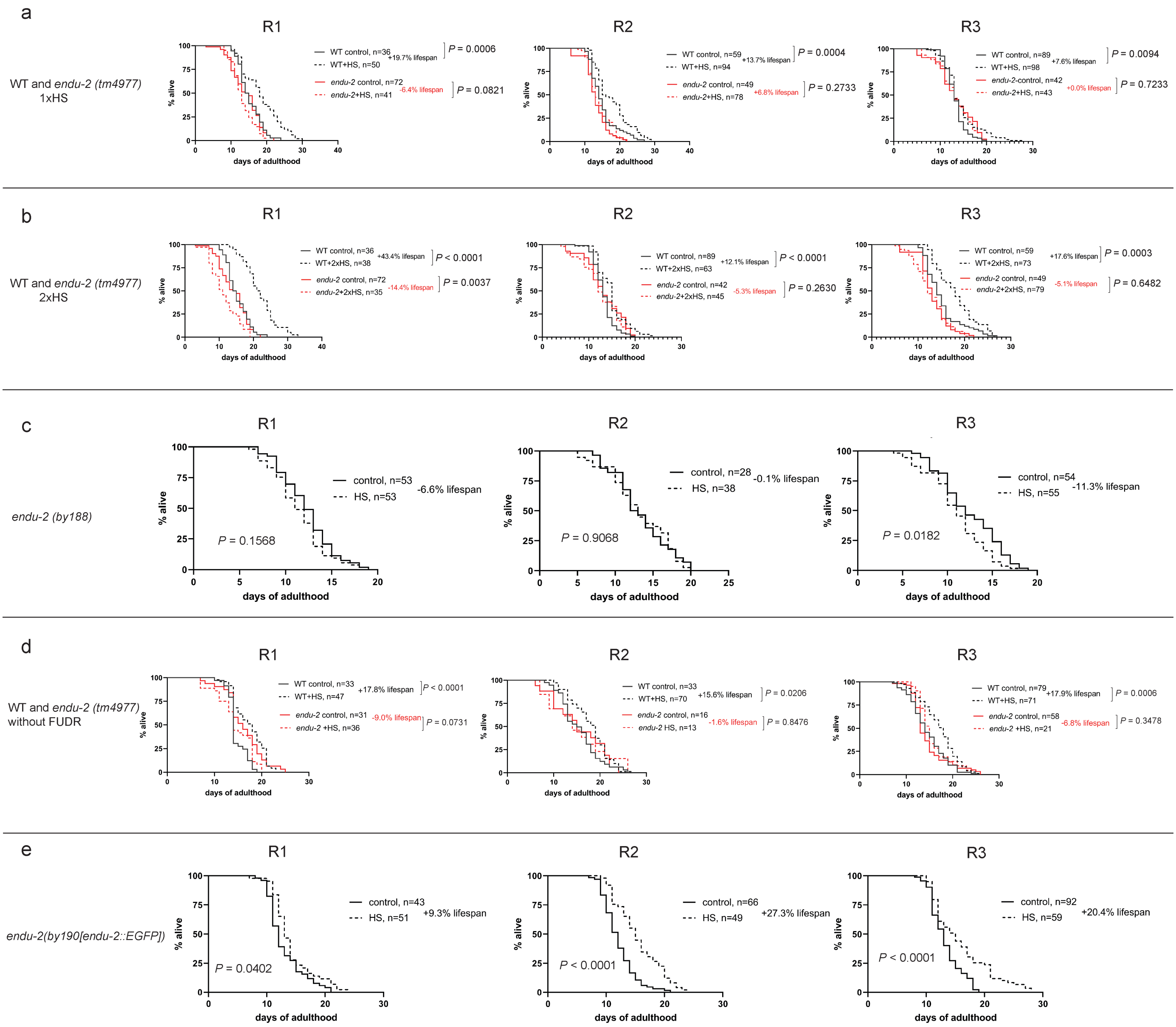

Supplementary Fig. 1 Summary of all biological replicates for the lifespan of WT and *endu-2* loss of function mutant animals

a, Lifespan analysis of WT and *endu-2(tm4977)* animals upon one hormetic HS treatment (1 h at 35°C) on day 1 of adulthood. **b,** Lifespan analysis of WT and *endu-2(tm4977)* animals upon hormetic HS on day 1 and day 3 of adulthood. **c,** Lifespan analysis of *endu-2(by188)* loss of function animals upon hormetic HS on day 1 and day 3 of adulthood. **d,** Lifespan analysis of WT and *endu-2(tm4977)* animals upon a 45-min HS at 36°C on day 1 of adulthood. The experiments were performed without FUDR. **e,** Lifespan analysis of *endu-2(by190[endu-2::EGFP])* CRISPR knock-in animals upon hormetic HS on day 1 and day 3 of adulthood. The *P* values were calculated using Log-rank (Mantel-Cox) test. This figure is related to **Fig. 1**.

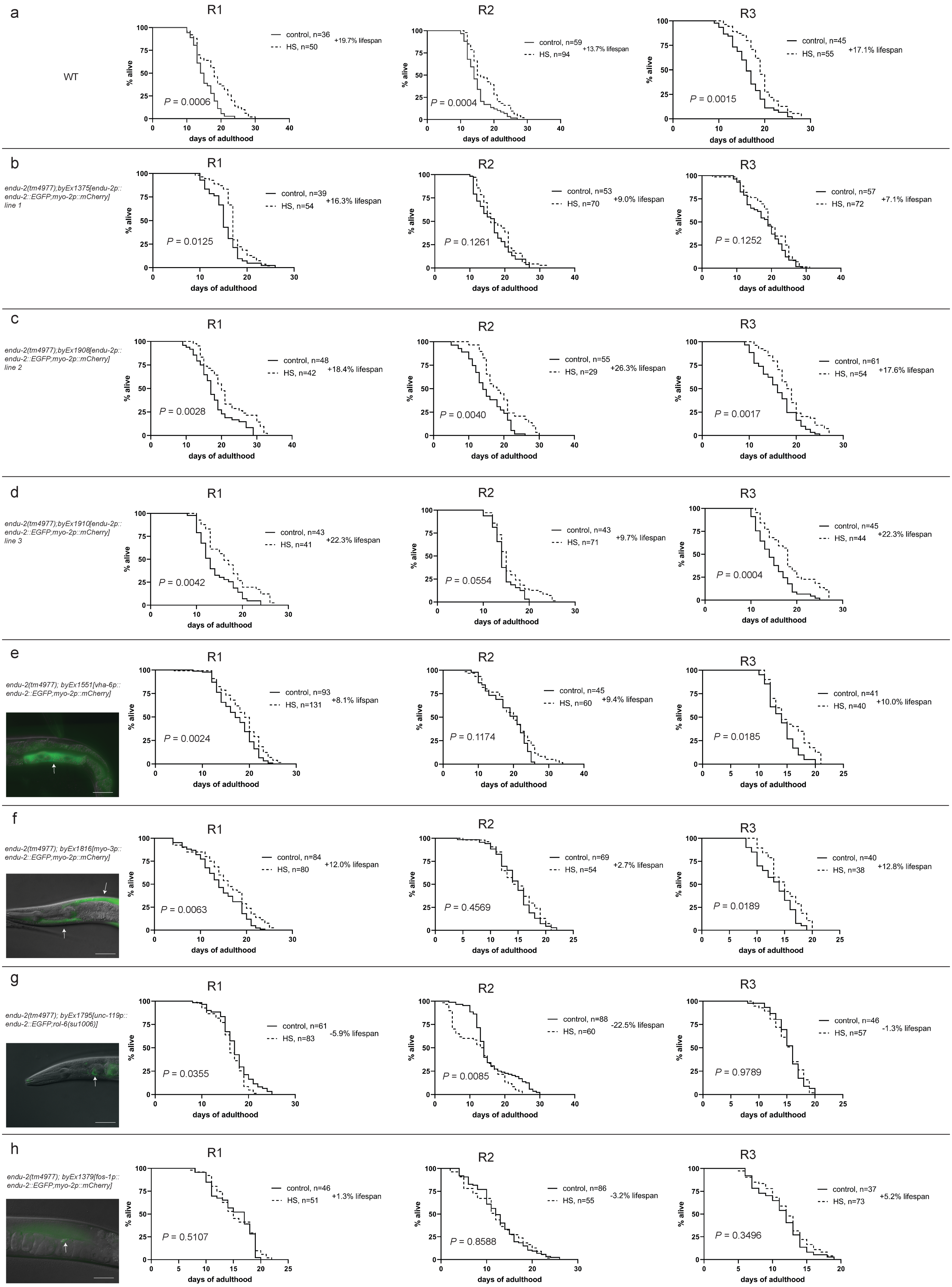

Supplementary Fig. 2 Summary of all biological replicates for Lifespan of WT and *endu-2::EGFP* rescued animals upon hormetic HS on day 1 and day 3 of adulthood.

a-h Lifespan analysis of WT and different *endu-2::EGFP* rescue strains upon 2x hormetic HS. The ENDU-2::EGFP fluorescence in each tissue-specific rescue strain is indicated with the white arrows. Scale bar = 50 µm. Shown are the three independent replicates. *P* values were calculated using Log-rank (Mantel-Cox) test. **a,** WT animals; **b,** *endu-2(tm4977);byEx1375[endu-2p::endu-2::EGFP]* (*endu-2::EGFP* rescue line 1) animals; **c,** *endu-2(tm4977);byEx1908[endu-2p::endu-2::EGFP]* (*endu-2::EGFP* rescue line 2) animals; **d,** *endu-2(tm4977);byEx1910[endu-2p::endu-2::EGFP]* (*endu-2::EGFP* rescue line 3) animals; **e,** *endu-2(tm4977);byEx1551[vha-6p::endu-2::EGFP::3xFlag]* (intestinal *endu-2* rescue); **f,** *endu-2(tm4977);byEx1816[myo-3p::endu-2::EGFP::3xFlag]* (muscular *endu-2* rescue); **g,** *endu-2(tm4977);byEx1795[unc-119p::endu-2::EGFP::3xFlag]* (neuronal *endu-2* rescue); **h,** *endu-2(tm4977);byEx1379[fos-1p::endu-2::EGFP::3xFlag]* (somatic gonadal *endu-2* rescue). This figure is related to **Fig. 1**.

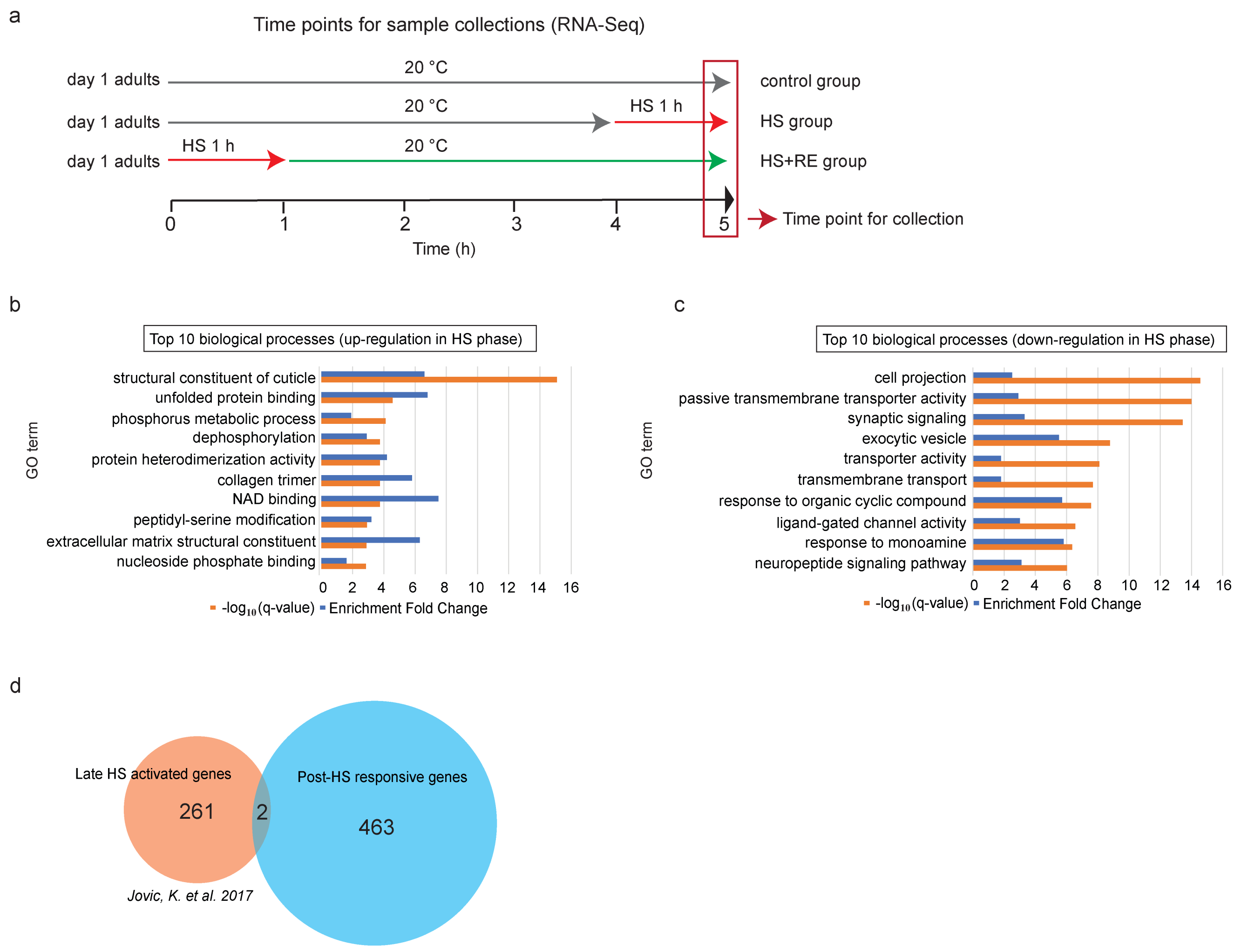

Supplementary Fig. 3 Gene Ontology (GO terms) enrichment analysis of DEGs upon HS.

a, Graphic illustration of stage matched sample collection for RNA-Seq. **b,** Top 10 most enriched GO terms of the activated genes upon 1 h HS in WT animals. **c,** Top 10 most enriched GO terms of the inactivated genes upon 1 h HS in WT animals. **d,** The Venn diagram shows the overlap between post-HS responsive genes and the late-responsive genes upon continuous HS. This figure is related to **Fig. 2**.

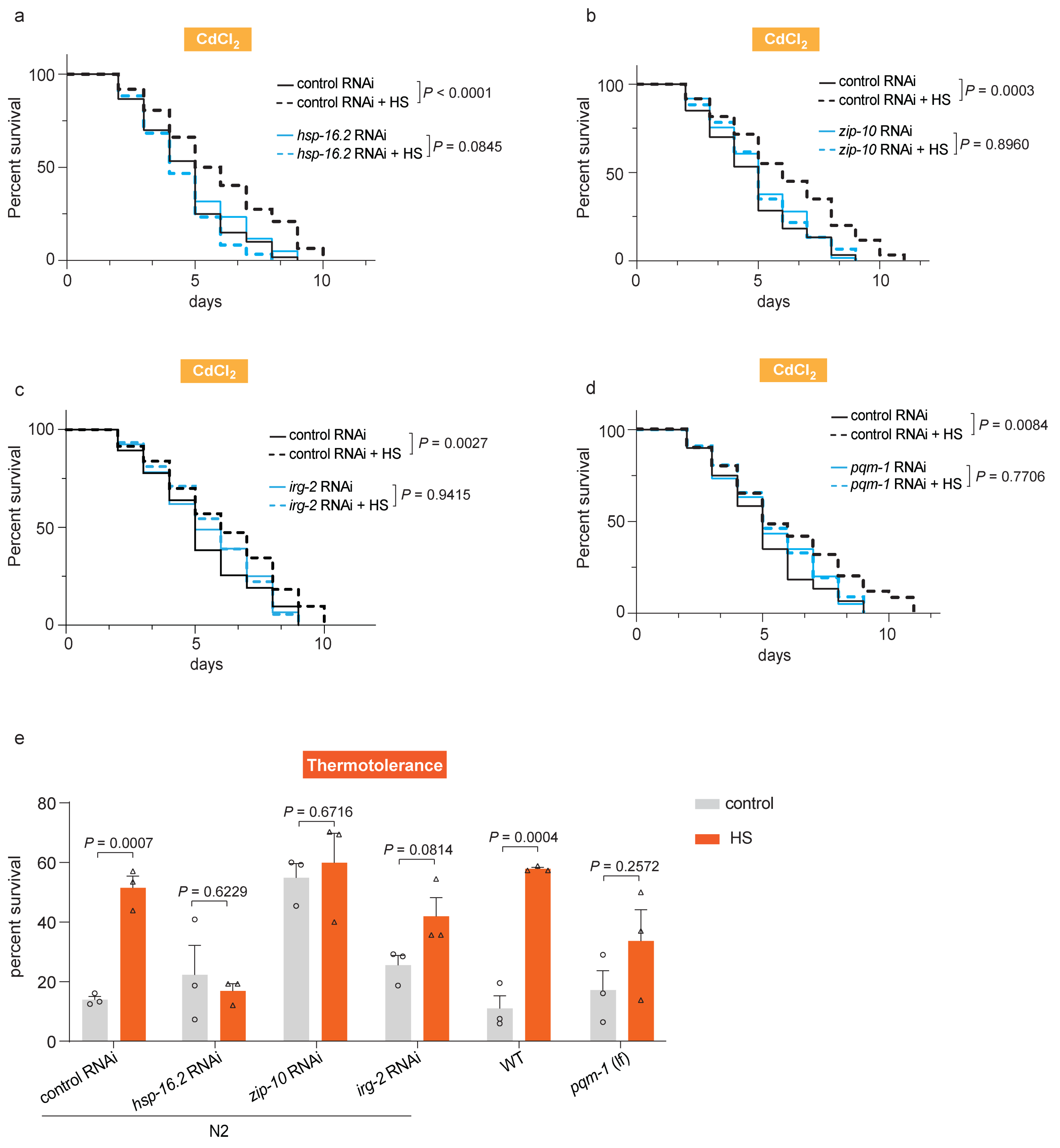

Supplementary Fig. 4 Activation of the post-HS responsive genes contributes to the beneficial effect of hormetic heat stress.

a-d, RNAi known-down of selected Class I (*hsp-16.2*), Class II (*zip-10*) and Class III (*irg-2* and *pqm-1*) post-HS responsive genes abolishes 1 h HS mediated beneficial effect against Cd^{2+} toxicity. Shown are pooled data from $N = 3$ independent experiments for all experiments. P values were calculated using Log-rank (Mantel-Cox) test.**a,** Mean survival of RNAi control without HS: 4.6 days, $n = 60$. RNAi control with HS: 6.2 days, $n = 60$. *hsp-16.2* RNAi knockdown without HS: 4.8 days, $n = 59$. *hsp-16.2* RNAi knockdown with HS: 4.9 days, $n = 61$. **b,** Mean survival of RNAi control without HS: 4.9 days, $n = 58$. RNAi control with HS: 6.6 days, $n = 60$. *zip-10* RNAi knockdown without HS: 5.2 days, $n = 29$. *zip-10* RNAi knockdown with HS: 5.2 days, $n = 60$. **c,** Mean survival of RNAi control without HS: 5.1 days, $n = 114$. RNAi control with HS: 6.3 days, $n = 113$. *irg-2* RNAi knockdown without HS: 5.4 days, $n = 112$. *irg-2* RNAi knockdown with HS: 5.6 days, $n = 120$. **d,** Mean survival of RNAi control without HS: 5.2 days, $n = 60$. RNAi control with HS: 6.3 days, $n = 58$. *pqm-1* RNAi knockdown without HS: 5.1 days, $n = 58$. *pqm-1* RNAi knockdown with HS: 5.4 days, $n = 62$. **e,** Thermotolerance of animals upon RNAi known-down of selected Class I (*hsp-16.2*), Class II (*zip-10*) and Class III (*irg-2* and *pqm-1*) animals after hormetic HS. For thermotolerance after hormetic heat stress, day one adult animals were incubated at 35°C for 1 h, followed by a 12 h recovery at 20°C before exposure to 35°C for 8 h. $N = 3$ for all experiments. Data are the mean ± SEM, P values were calculated with two-tailed multiple unpaired t-test. This figure is related to **Fig. 2**.

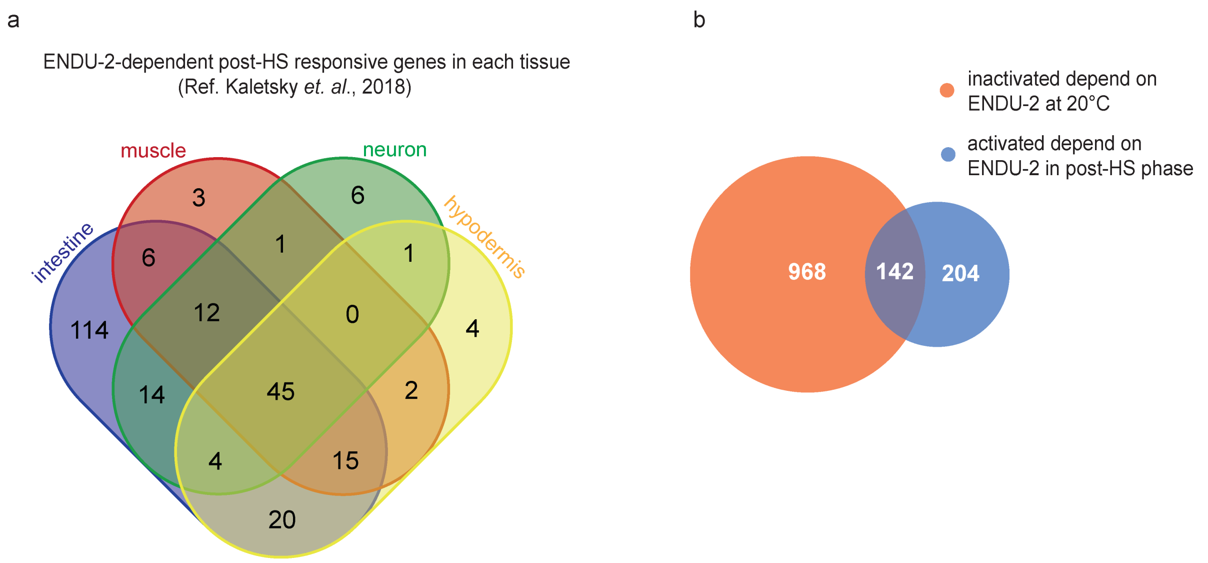

Supplementary Fig. 5 Intestine-only genes are dominant in the ENDU-2 dependent post-HS responsive genes

a, Venn diagram displaying the overlap between ENDU-2 dependent post-HS responsive genes and genes are expressed in each somatic tissue (intestine, muscle, neuron and hypodermis). **b,** Venn diagram displaying the overlap between genes inactivated by ENDU-2 at 20°C and genes activated by ENDU-2 in the post-HS phase. This figure is related to **Fig. 2**.

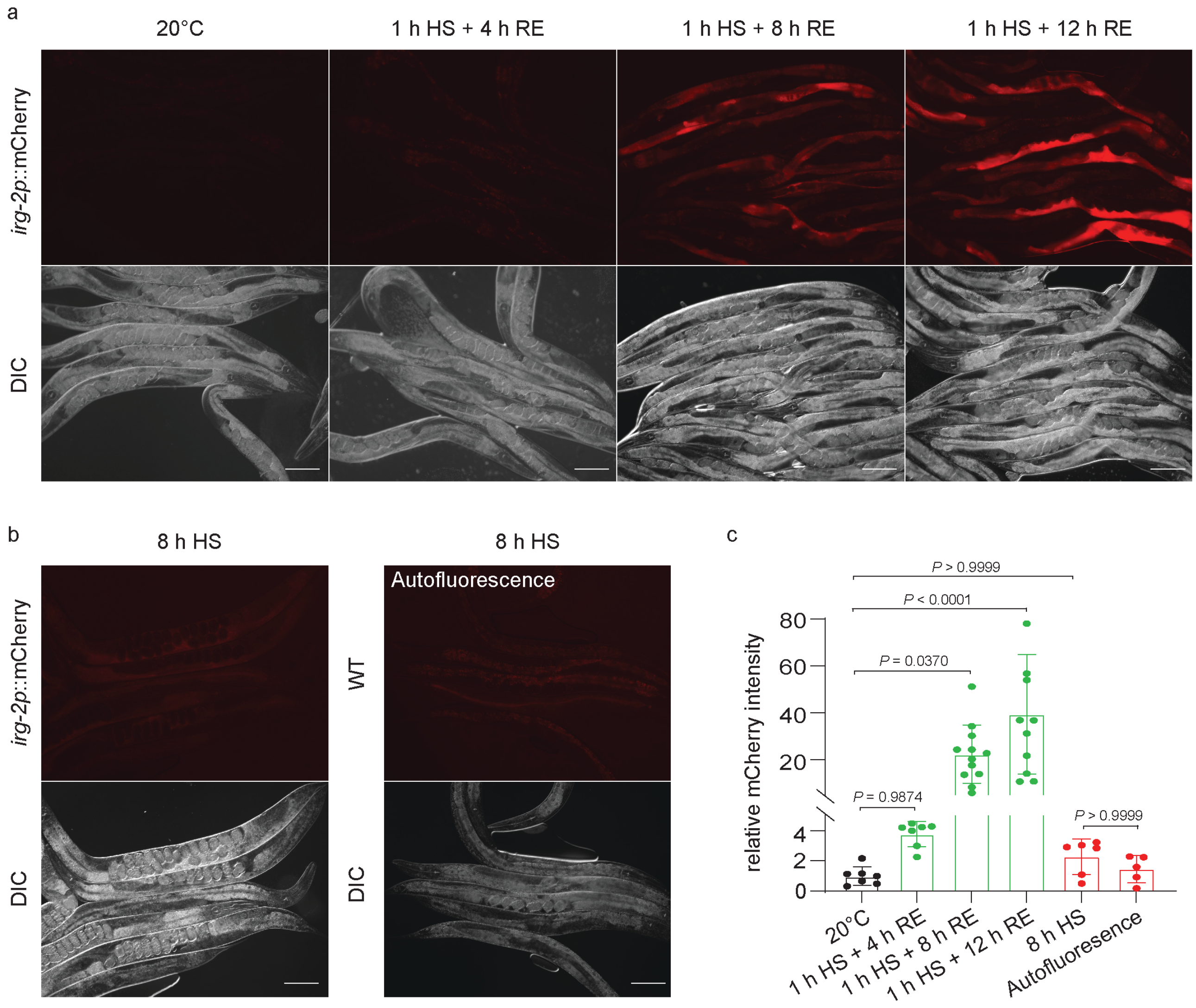

Supplementary Fig. 6 Transcriptional activation of *irg-2* occurs specifically in the post-HS phase.

a, Fluorescence micrographs of *byIs296[irg-2p::mCherry]* transgenic animals under indicated conditions. Scale bar = 100 μm. **b,** Fluorescence micrographs of *byIs296[irg-2p::mCherry]* transgenic animals upon 8 h continuous HS. Fluorescence in WT N2 animals treated under the same condition serves as a negative control to exclude possible interference of gut autofluorescence in mCherry signal. Scale bar = 100 μm. **c,** Quantification of the relative fluorescence intensity of mCherry in *byIs296[irg-2p::mCherry]* transgenic animals under the indicated conditions in the **(a)** and **(b)**. Transgenic animals at control (20°C): n = 7.

Transgenic animals with 1 h HS plus 4 h recovery: $n = 7$. Transgenic animals with 1 h HS plus 8 h recovery: $n = 12$. Transgenic animals with 1 h HS plus 12 h recovery: $n = 10$. Transgenic animals with 8 h HS without recovery: $n = 6$. WT animals with 8 h HS without recovery: $n = 5$. Data are the mean ± SD, P-values were calculated using one-way ANOVA with Tukey's multiple comparisons test. This figure is related to **Fig. 3**.

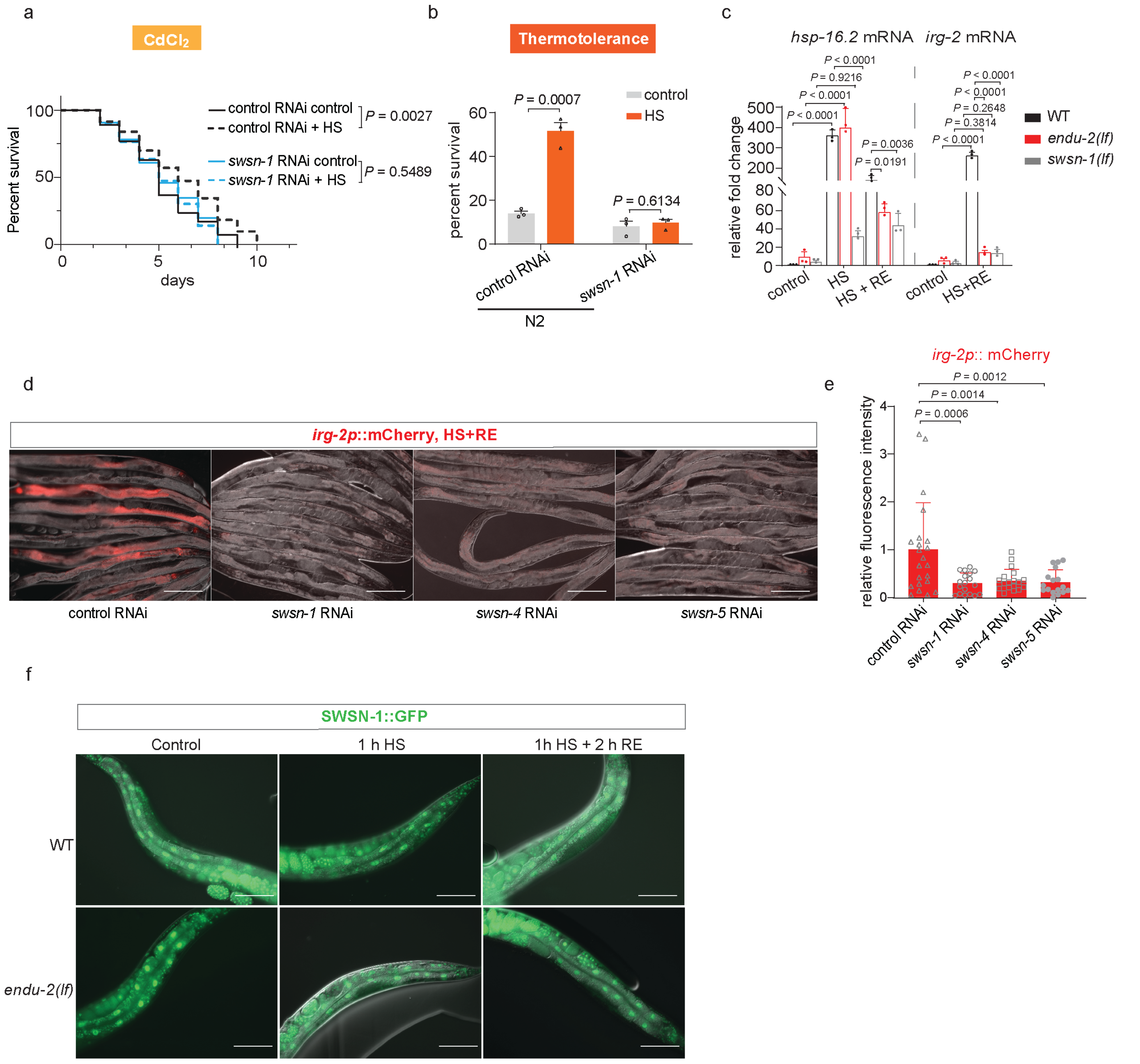

Supplementary Fig. 7 SWI/SNF nucleosome remodeling complex affects both HS and post-HS responses to mediate heat hormesis.

a, RNAi knock-down of *swsn-1* abolishes hormetic HS mediated protective effect against Cd^{2+} toxicity. Mean survival of RNAi control without HS: 5.2 days, $n = 94$. RNAi control with HS: 6.1 days, $n = 93$. *swsn-1* RNAi

without HS: 5.3 days, *n* = 87. *swsn-1* RNAi with HS: 5.2 days, *n* = 93. Shown are pooled data of *N* = 3 independent experiments. *P* values were calculated using Log-rank (Mantel-Cox) test. **b,** RNAi knock-down of *swsn-1* abolishes improved thermotolerance by hormetic HS in WT animals. For thermotolerance after hormetic heat stress, day one adult animals were incubated at 35°C for 1 h, followed by a 12 h recovery at 20°C before exposure to 35°C for 8 h. Survival rates for each group: WT with control RNAi without HS: (13.9 ± 3.2) %, *n* = 92. WT with control RNAi with HS: (51.5 ± 5.5) %, *n* = 97. WT with *swsn-1* RNAi without HS: (8.1 ± 4.2) %, *n* = 111. WT with *swsn-1* RNAi with HS: (9.6 ± 3.5) %, *n* = 107. *N* = 3 for all groups. Data are the mean ± SEM, *P* values were calculated with two-tailed multiple unpaired t-test. **c,** qRT-PCR quantification of relative *hsp-16.2* and *irg-2* mRNA levels of the representative post-HS responsive genes in WT, *endu-2(tm4977)* and *swsn-1(os22)* animals in indicated conditions. *N* = 3 independent experiments. Data are the mean ± SD. *P*-values were calculated using two-way ANOVA with Tukey's multiple comparisons test. **d,** Fluorescence micrographs of *byIs296[irg-2p::mCherry]* transgenic animals under RNAi known-down of *swsn-1, swsn-4* and *swsn-5* in post-HS phase. Scale bar = 100 µm. **e,** Quantification of the relative fluorescence intensity of mCherry in *byIs296[irg-2p::mCherry]* transgenic animals under the indicated condition in the **(d)**. RNAi control: *n* = 24. *swsn-1* RNAi knock down: *n* = 18. *swsn-4* RNAi knock down: *n* = 21. *swsn-5* RNAi knock down: *n* = 17. Data are the mean ± SD, *P*-values were calculated using one-way ANOVA with Tukey's multiple comparisons test. **f,** SWSN-1::GFP protein is localized in the nucleus independent of ENDU-2. Shown are fluorescence micrographs of *st12187[swsn-1::TY1::EGFP::3xFLAG]* animals in WT or *endu-2(tm4977)* background. SWSN-1::GFP was detected in day 1 adult animals subjected to 1 h HS or 1 h HS followed by 2 h recovery at 20°C. *N* = 3 independent experiments. Scale bar = 50 µm. This figure is related to **Fig. 3**.

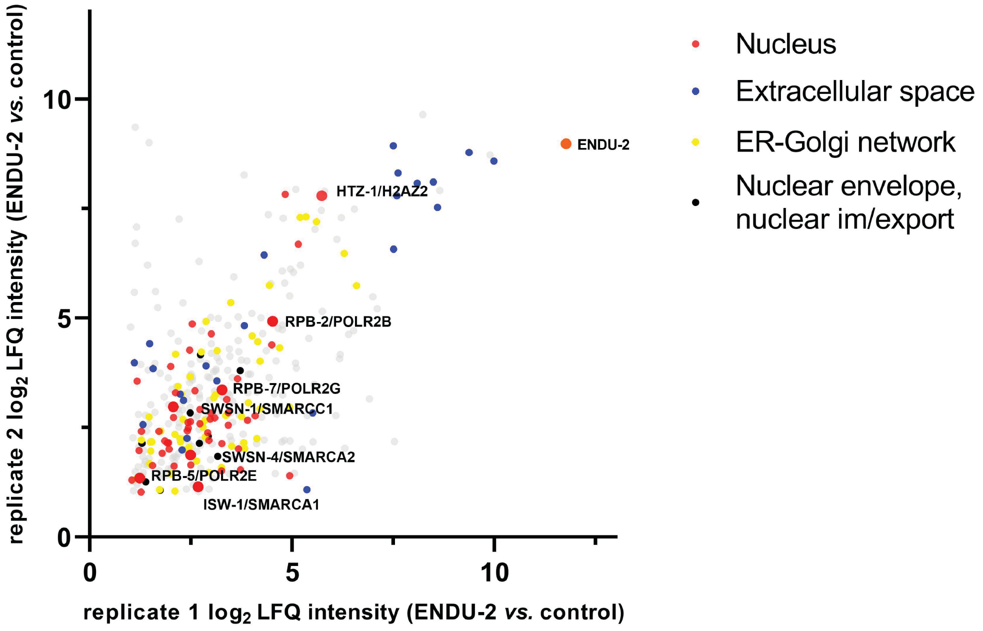

Supplementary Fig. 8 Scatter Plot comparing two biological replicates of ENDU-2 MS interactor analysis

ENDU-2 (orange dot) associates with multiple proteins affecting in ER-Golgi transport (yellow dots), proteins in the extracellular matrix (blue dots), nuclear proteins (red dots) as well as factors affecting nuclear import/export (black dots) (log_2LFD intensity > 1). In the plot, histone protein HTZ-1, components of SWI/SNF complex: SWSN-1, SWSN-4 and ISW-1, and three subunits of RNA polymerase II (Pol II) (RPB-2, RPB-5 and RPB-7) are highlighted by larger red dots. This figure is related to **Fig. 4**.

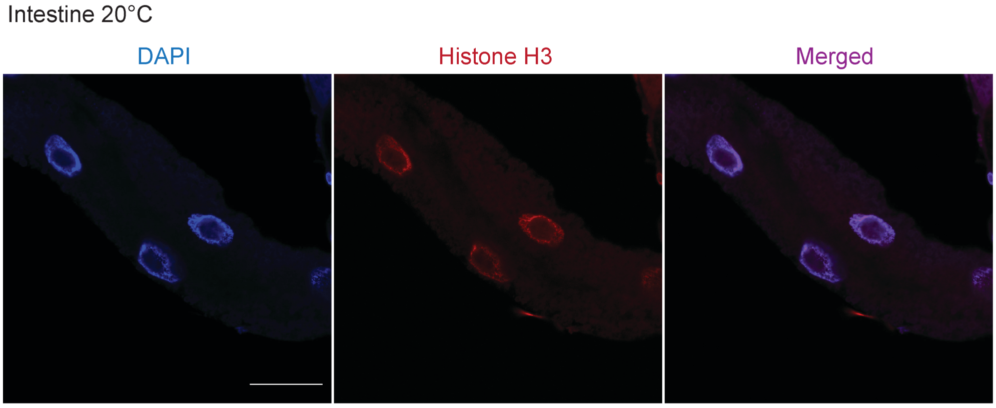

Supplementary Fig. 9 Histone H3 colocalizes with the DAPI-stained DNA

Immunofluorescence staining of histone H3 in *endu-2(by190[endu-2::EGFP])* CRISPR knock-in animals at 20°C. Scale bar = 20 µm. *N* = 2 independent experiments. This figure is related to **Fig. 4**.

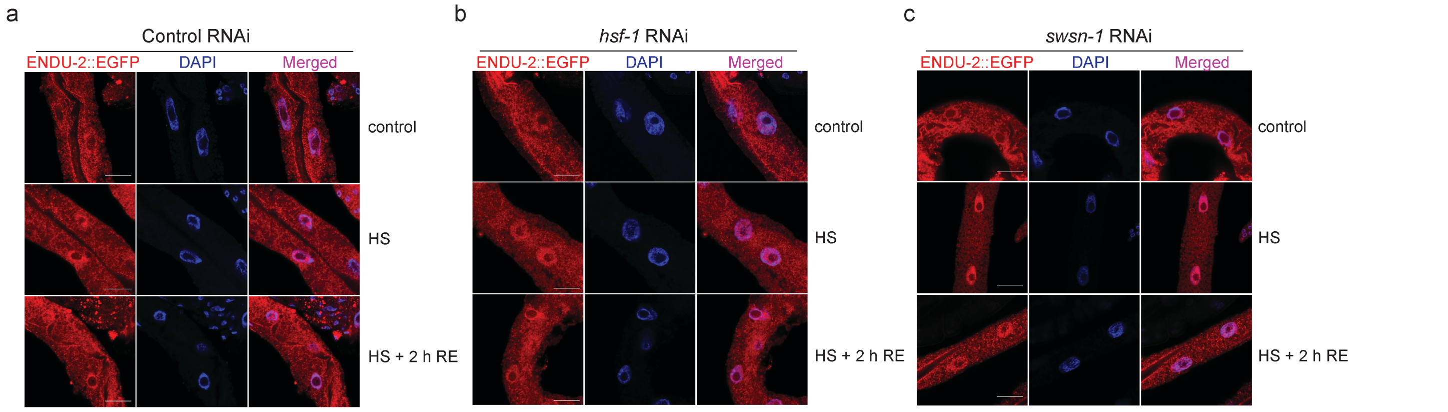

Supplementary Fig. 10 RNAi knock-down of neither *hsf-1* nor *swsn-1* impairs chromatin localization of ENDU-2 during and after HS

a-c, Immunofluorescence staining of ENDU-2::EGFP with GFP antibody in *endu-2(by190[endu-2::EGFP])* animals upon *hsf-1* or *swsn-1* RNAi and subjected to 1 h HS or 1 h HS followed by 2 h recovery. Scale bar = 20 μm. *n* = 6 for each condition. This figure is related to **Fig. 4**.

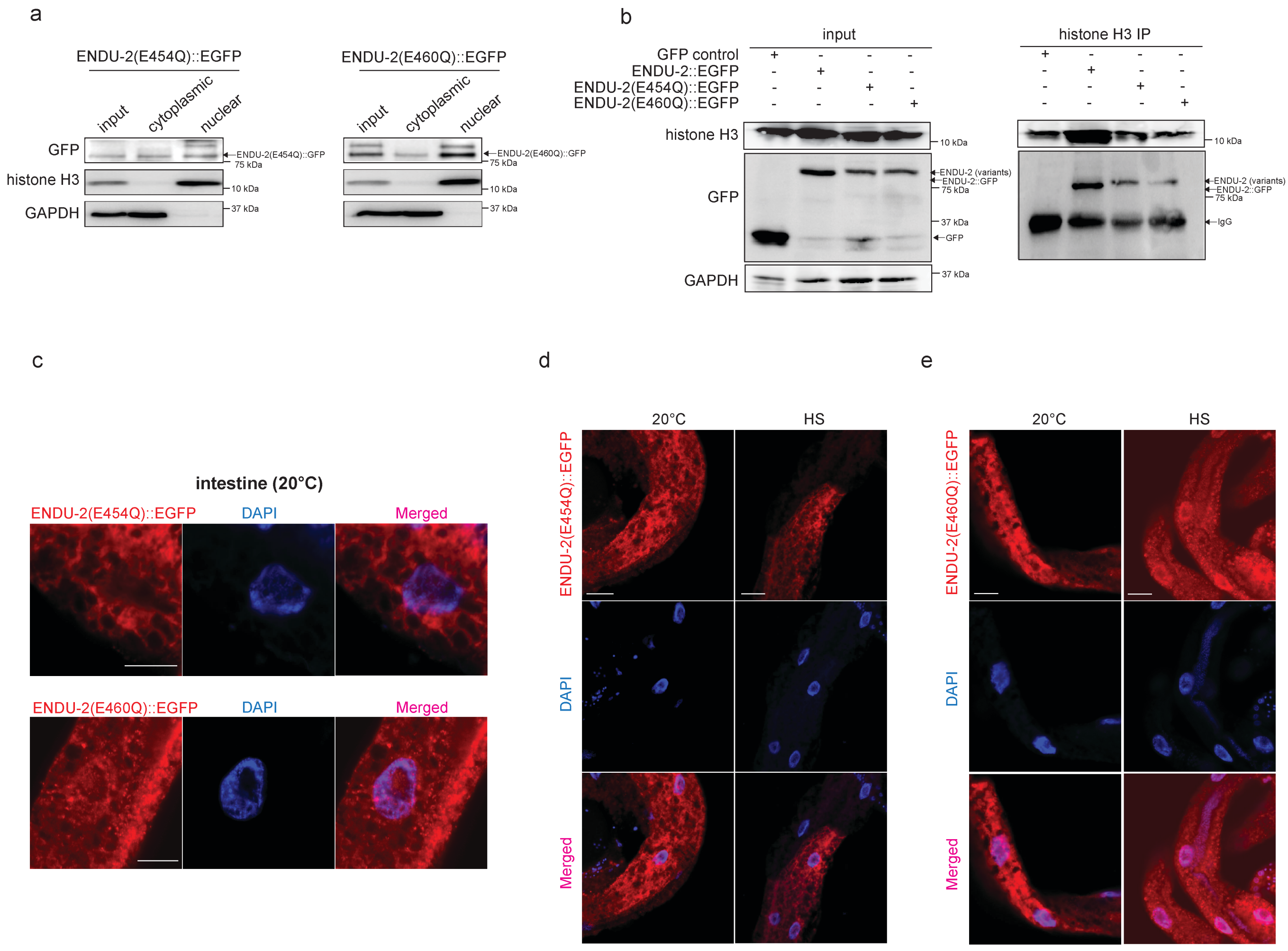

Supplementary Fig. 11 ENDU-2 mutant variants show different subcellular localization

a, Western Blot detection of ENDU-2(E454Q)::EGFP and ENDU-2(E460Q)::EGFP in different subcellular fractions. ENDU-2(E454Q)::EGFP and ENDU-2(E460Q)::EGFP are detected in whole cell lysis (input), cytoplasmic fraction and nuclear fraction. Histone H3 and GAPDH serve as controls for nuclear and cytoplasmic fractions, respectively. *N* = 3 independent experiments. **b,** ENDU-2(E454Q)::EGFP and ENDU-2(E460Q)::EGFP is co-immunoprecipitated with histone H3, respectively. A transgenic *Is[sod-3p::gfp]* strain serves as a negative control to exclude the interaction between GFP and histone H3. *N* = 3 independent experiments. **c,** Immunofluorescence staining of ENDU-2(E454Q)::EGFP and ENDU-2(E460Q)::EGFP with GFP antibody. The upper panel shows that ENDU-2(E454Q)::EGFP is diffusely distributed both in the cytoplasm and nucleus. The lower panel shows that ENDU-2 (E460Q)::EGFP is formed into puncta structures in addition to diffused distribution both in cytoplasm and nuclei. Scale bar = 10 µm. $n > 20$ for each group. *N* = 3 independent experiments. **d,** HS does not significantly alter the subcellular localization of ENDU-2(E454Q)::EGFP. Shown are immunofluorescence of unstressed and 1 h HS stressed ENDU-2(E454Q)::EGFP day 1 adult animals stained with GFP antibody. Scale bar = 20 µm. $n = 5$ for unstressed condition, $n = 17$ for HS. **e,** HS enhances chromatin localization of ENDU-2(E460Q)::EGFP. Shown are immunofluorescence of unstressed and 1 h heat stressed ENDU-2(E460Q)::EGFP day 1 adult animals stained with GFP antibody. Scale bar = 20 µm. $n = 13$ unstressed condition, $n = 5$ for HS. This figure is related to **Fig. 5**.

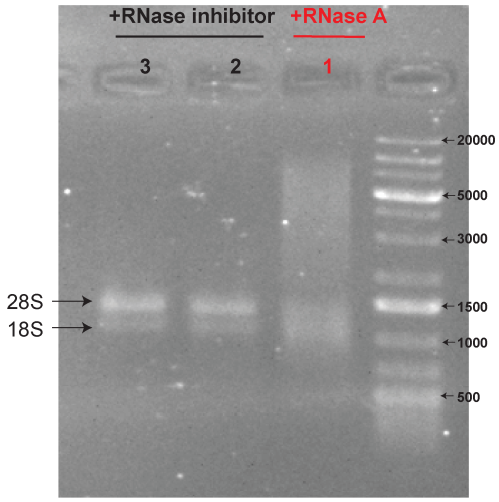

Supplementary Fig. 12 Analyzing RNA integrity after treatment with RNase A or RNase inhibitor

1% TAE agarose gels containing 1% Sodium Hypochlorite (Carl Roth) was used for RNA electrophoresis. The absence of 28S, 18S ribosomal RNA (rRNA) bands indicates successful RNA decay with RNase A treatment (lane 1). Intact rRNA bands in the RNase inhibitors treated samples (lanes 2 and 3) indicate preservation of RNA integrity for ENDU-2/AMA-1 co-IP experiment. *N* = 3 independent experiments. This figure is related to **Fig. 6**.
