# Supplementary material for: Reprogramming of the transcriptome after heat stress mediates heat hormesis in Caenorhabditis elegans
Source: Nat Commun. 2023 Jul 13;14:4176. doi: 10.1038/s41467-023-39882-8 (PMC10345090; doi:10.1038/s41467-023-39882-8)
Supplement: Supplementary file 3 — Description of Additional Supplementary Files [file 41467_2023_39882_MOESM3_ESM.pdf]

Description of Additional Supplementary Files

Supplementary Data 1: Differentially expressed genes in WT or endu-2(tm4977) animals.

Supplementary Data 2: Differentially expressed genes are classified from post-HS responsive genes (heat map).

Supplementary Data 3: ENDU-2-dependent genes in different conditions.

Supplementary Data 4: ENDU-2-dependent post-HS responsive genes in each tissue.

Supplementary Data 5: list of proteins co-purifying with ENDU-2::EGFP (log2LFD intensity > 1).

Supplementary Data 6: Strains were used for this study.

Supplementary Data 7: Primers information in this study.
