# Supplementary material for: Reprogramming of the transcriptome after heat stress mediates heat hormesis in Caenorhabditis elegans
Source: Nat Commun. 2023 Jul 13;14:4176. doi: 10.1038/s41467-023-39882-8 (PMC10345090; doi:10.1038/s41467-023-39882-8)
Supplement: Supplementary file 11 — Reporting Summary [file 41467_2023_39882_MOESM11_ESM.pdf]

nature portfolio

Corresponding author(s): Wenjing Qi

Last updated by author(s): Jun 5, 2023

Reporting Summary

Nature Portfolio wishes to improve the reproducibility of the work that we publish. This form provides structure for consistency and transparency in reporting. For further information on Nature Portfolio policies, see our Editorial Policies and the Editorial Policy Checklist.

Statistics

For all statistical analyses, confirm that the following items are present in the figure legend, table legend, main text, or Methods section.

n/a	Confirmed	
[ ]	[x]	The exact sample size (*n*) for each experimental group/condition, given as a discrete number and unit of measurement
[ ]	[x]	A statement on whether measurements were taken from distinct samples or whether the same sample was measured repeatedly
[ ]	[x]	The statistical test(s) used AND whether they are one- or two-sided<br>*Only common tests should be described solely by name; describe more complex techniques in the Methods section.*
[ ]	[x]	A description of all covariates tested
[ ]	[x]	A description of any assumptions or corrections, such as tests of normality and adjustment for multiple comparisons
[ ]	[x]	A full description of the statistical parameters including central tendency (e.g. means) or other basic estimates (e.g. regression coefficient) AND variation (e.g. standard deviation) or associated estimates of uncertainty (e.g. confidence intervals)
[ ]	[x]	For null hypothesis testing, the test statistic (e.g. *F*, *t*, *r*) with confidence intervals, effect sizes, degrees of freedom and *P* value noted<br>*Give P values as exact values whenever suitable.*
[x]	[ ]	For Bayesian analysis, information on the choice of priors and Markov chain Monte Carlo settings
[x]	[ ]	For hierarchical and complex designs, identification of the appropriate level for tests and full reporting of outcomes
[x]	[ ]	Estimates of effect sizes (e.g. Cohen's *d*, Pearson's *r*), indicating how they were calculated

Our web collection on statistics for biologists contains articles on many of the points above.

Software and code

Policy information about availability of computer code

Data collection	No
Data analysis	1.mRNA sequencing analysis was carried out with the online galaxy europe (http://usegalaxy.eu/). FastQC (Galaxy Version 0.72+galaxy1), RNA STAR (Galaxy Version 2.7.8a), featureCounts(Galaxy Version 2.0.1) and DESeq2 R package (Galaxy Version 2.11.40.6+galaxy2).<br>2.GO term enrichment analysis was carried out with the online enrichment analysis tool (https://wormbase.org/tools/enrichment/tea/tea.cgi)<br>3.Statistical analysis was performed on the GraphPad Prism 9.3.1 (471) software.<br>4. Fluorescence of individual worms was processed with Carl Zeiss ZEN 2 (blue edition) and quantified using Fiji image J.<br>5. Graphs were made by GraphPad Prism 9.3.1 (471) software.<br>6. Figures were made by Adobe Illustrator 2021.<br>7. Western Blot results were processed and analyzed with Multi Gauge V3.0.

For manuscripts utilizing custom algorithms or software that are central to the research but not yet described in published literature, software must be made available to editors and reviewers. We strongly encourage code deposition in a community repository (e.g. GitHub). See the Nature Portfolio guidelines for submitting code & software for further information.

Data

Policy information about availability of data

All manuscripts must include a data availability statement. This statement should provide the following information, where applicable:

- Accession codes, unique identifiers, or web links for publicly available datasets
- A description of any restrictions on data availability
- For clinical datasets or third party data, please ensure that the statement adheres to our policy

The RNA-seq data are deposited in BioProject under BioProject ID PRJNA881926. The proteomics data are available via ProteomeXchange with identifier PXD041872. Source data are provided with this paper.

Research involving human participants, their data, or biological material

Policy information about studies with human participants or human data. See also policy information about sex, gender (identity/presentation), and sexual orientation and race, ethnicity and racism.

Reporting on sex and gender	No
Reporting on race, ethnicity, or other socially relevant groupings	No
Population characteristics	No
Recruitment	No
Ethics oversight	No

Note that full information on the approval of the study protocol must also be provided in the manuscript.

Field-specific reporting

Please select the one below that is the best fit for your research. If you are not sure, read the appropriate sections before making your selection.

☒ Life sciences ☐ Behavioural & social sciences ☐ Ecological, evolutionary & environmental sciences

For a reference copy of the document with all sections, see nature.com/documents/nr-reporting-summary-flat.pdf

Life sciences study design

All studies must disclose on these points even when the disclosure is negative.

Sample size	We chose sample sizes that have been wildly accepted in the field for phenotype analysis .<br>For mRNA Sequencing we performed two independent experiment for extracting mRNAs from wide type and endu-2 loss of function mutant animals, respectively.<br>For mass spectrometry, two independent immunoprecipitation of ENDU-2 endougenous protein and control.
Data exclusions	No
Replication	All attempts at replication were successful.
Randomization	Samples were allocated randomly into experimental groups.
Blinding	All experiment were blinded to group allocation during data collection.

Reporting for specific materials, systems and methods

We require information from authors about some types of materials, experimental systems and methods used in many studies. Here, indicate whether each material, system or method listed is relevant to your study. If you are not sure if a list item applies to your research, read the appropriate section before selecting a response.

Materials & experimental systems

n/a	Involved in the study
☐	☒ Antibodies
☒	☐ Eukaryotic cell lines
☒	☐ Palaeontology and archaeology
☐	☒ Animals and other organisms
☒	☐ Clinical data
☒	☐ Dual use research of concern
☒	☐ Plants

Methods

n/a	Involved in the study
☒	☐ ChIP-seq
☒	☐ Flow cytometry
☒	☐ MRI-based neuroimaging

Antibodies

Antibodies used

1. anti-GFP (Roche, Catalog: Nr. 11814460001, Monoclonal mouse clones 7.1 and 13.1);
2. Goat anti-Mouse IgG (H+L) Cross-Adsorbed Secondary Antibody, Alexa Fluor™ 555 (Invitrogen, Catalog: # A-21422, Polyclonal);
3. anti-Pol II 8WG16 (Santa Cruz Biotechnology, Catalog: sc-56767, mouse monoclonal IgG 2a κ);
4. anti-POLR2B (RBP-2) (Invitrogen, Catalog: # PA5-30122, Rabbit polyclonal);
5. anti-GFP (abcam, Catalog: ab290, Rabbit polyclonal).
6. anti-histone H3 (abcam, Catalog: ab1791, Rabbit polyclonal).
7.HRP-Goat anti-Rabbit IgG (H+L) Secondary Antibody (Invitrogen, Catalog: #31460)
8. HRP-Goat anti-mouse IgG (H+L) Secondary Antibody (Invitrogen, Catalog: #31430)

Validation

1. Monoclonal mouse anti-GFP antibody(clones 7.1 and 13.1) for detection of both wild-type and mutant forms of GFP or GFP fusions (https://www.sigmaaldrich.com/DE/en/product/roche/);
2. goat anti-mouse Alexa Fluor 555 against bovine IgG, goat IgG, rabbit IgG, rat IgG, human IgG and human serum, Anti-Mouse secondary antibodies are affinity-purified antibodies with well-characterized specificity for mouse immunoglobulins and are useful in the detection, sorting or purification of its specified target. (https://www.thermofisher.com/antibody/product/Goat-anti-Mouse-IgG-H-L-Highly-Cross-Adsorbed-Secondary-Antibody-Polyclonal/A32727);
3. anti-Pol II antibody (8WG16) is recommended for the detection of the highly conserved heptapeptide repeat of the largest subunit of eukaryotic Pol II from the species mouse, rat, human, Xenopus, C. elegans, yeast, wheat germ and bovine by WB , IP and IF (https://www.scbt.com/p/pol-ii-antibody-8wg16);
4. recombinant fragment corresponding to a region within amino acids 905 and 1123 of Human RPB2 (https://www.thermofisher.com/antibody/product/POLR2B-Antibody-Polyclonal/PA5-30122);
5. RTypically they tolerate N- and C-terminal fusion to a broad variety of proteins. They have been expressed in most known cell types and are used as a noninvasive fluorescent marker in living cells and organisms. They enable a wide range of applications where they have functioned as a cell lineage tracer, reporter of gene expression, or as a measure of protein-protein interactions. (https://www.abcam.com/gfp-antibody-ab290.html).
6. Reacts with: Mouse, Rat, Human, Saccharomyces cerevisiae, Xenopus laevis, Arabidopsis thaliana, Drosophila melanogaster, Indian muntjac, Schizosaccharomyces pombe(https://www.abcam.com/products/primary-antibodies/histone-h3-antibody-nuclear-marker-and-chip-grade-ab1791.html).

Animals and other research organisms

Policy information about studies involving animals; ARRIVE guidelines recommended for reporting animal research, and Sex and Gender in Research

Laboratory animals

The strain name use in this study:
N2
BR6961
BR7295
BR8662
BR7827
BR7332
BR8551
TJ375
HS304
BR7205
CF1553
BR7130
BR7680
BR7683
BR8352
BR7803
BR7802
BR8850

	BR8976<br>BR8827<br>BR8977<br>BR8995<br>BR9215<br>BR9217<br>BR8994<br>BR9143<br>all the animals for the experiments are hermaphrodites, one-day-old animals.
Wild animals	No
Reporting on sex	No
Field-collected samples	Strains were maintained at 15°C and using Escherichia coli (E. coli) OP50 as a food source. Animals used for experiment were cultured at 20°C from embryonic stage.
Ethics oversight	No ethical approval was required as all the experiments were performed in C. elegans

Note that full information on the approval of the study protocol must also be provided in the manuscript.
